# Correction: Enhancement of oral bioavailability of ibrutinib using a liposil nanohybrid delivery system

**DOI:** 10.1371/journal.pone.0315777

**Published:** 2024-12-11

**Authors:** The PLOS ONE Staff

The funding statement for this paper is incorrect. The publisher apologizes for this error. The correct statement is: The authors extend their appreciation to the Deanship of Research and Graduate Studies at King Khalid University for funding this work through Small group Research Project under grant number RGP1/136/45.

The following Acknowledgements is missing from the article: The authors extend their appreciation to the Deanship of Research and Graduate Studies at King Khalid University for funding this work through Small Group Research Project under grant number RGP1/136/45.
