# Supplementary material for: Enhancement of oral bioavailability of ibrutinib using a liposil nanohybrid delivery system
Source: PLoS One. 2024 Sep 24;19(9):e0310492. doi: 10.1371/journal.pone.0310492 (PMC11421787; doi:10.1371/journal.pone.0310492)
Supplement: Attachment — Submitted filename: Response to reviewer comments.docx [file pone.0310492.s001.docx]

**Response to reviewer comments**

**Reviewer 1**

| **Serial No.** | **Reviewer Comment** | **Response** |
| --- | --- | --- |
| 1 | Please check the grammar throughout the manuscript | The grammar has been improved throughout the manuscript as suggested by the reviewer. |
| 2 | Do the authors expect silicosis due to the silica coating? | Authors are not expecting that these liposils can cause silicosis as the concentration of the materials used for the silica coating is much less and is meant for oral use only. Silicosis is a condition that results from the excessive inhalation of silica containing dust. |
| 3 | Please specify the type of DLS equipment applied for the measurements. | The DLS make and model are included in the **2.5.1** section of the manuscript. |
| 4 | Please specify the parameters of the disc prepared for FTIR! (Diameter, applied compression force etc.)! | The suggested parameters are included in the manuscript in the **2.5.4.1** section of the manuscript. |
| 5 | What was the reason for using 1M sodium salicylate in the release medium? Please explain! | 1M sodium salicylate in the release medium ensures sink conditions, enhances solubility, stabilizes the drug, and may improve analytical sensitivity, enabling accurate assessment of dissolution behavior. |
| 6 | The nanoliposomes showed positive zeta potential. Can this have an effect on the absorption? | Yes, the zeta potential of nanoliposomes can potentially affect their absorption properties, especially in biological systems or when considering interactions with other particles or surfaces. In terms of absorption, the zeta potential of nanoliposomes could affect their ability to adhere to or be taken up by cells, tissues, or biological barriers. Positively charged nanoparticles may have enhanced interactions with negatively charged cell membranes due to electrostatic attraction, potentially leading to increased cellular uptake or absorption. |
| 7 | Besides the drug encapsulation efficacy (EE) the drug loading (DL) is also an important parameter. Please provide it! | The data pertaining to drug loading is already provided in the manuscript. |
| 8 | Based on the DSC and XRPD results the drug went through amorphization. How can we be sure, that the drug is inside the nanoparticle? | From the FTIR results, the characteristic peaks of ibrutinib in both formulations were shifted, indicating the encapsulation of ibrutinib in liposomes. |
| 9 | In case of Fig. 2A what does CM mean on the x-scale? Usually, wavenumber is plotted on the x-scale of FTIR curve. | In Figure **2A,** on the x-scale wavenumber is now updated as “Wavenumber (cm^-1^)” on the x-axis. |
| 10 | Standard deviations are missing from the Figures! I guess there were more parallel experiments performed. | Parallel Experiments were performed and the Average values are utilized for the data plotting in the entire study |
| 11 | What is the target concentration of ibrutinib? In the MTT studies it is visible, that above 10 µM the formulations show cytotoxicity. | The target concentration of ibrutinib, as inferred from the MTT studies, appears to be below 10 µM. This conclusion is drawn from the observed cytotoxicity of the formulations at concentrations above this threshold.  The MTT assay results indicate that the inhibitory ratios of the formulations are dose-dependent, with decreasing cell viability as the concentration of ibrutinib increases. Specifically, the IC_50_ values, representing the concentration of ibrutinib required to inhibit 50% of cell viability, were found to be least for the free ibrutinib formulation at 23.45 ± 1.37 μM. However, for the formulations containing ibrutinib encapsulated within liposils and nanoliposomes, higher IC_50_ values were observed, indicating reduced cytotoxicity compared to the free drug. These values were measured at 44.56 ± 2.43 μM and 53.19 ± 3.12 μM, respectively.  The noticeable cytotoxic effects observed above 10 µM suggest that concentrations below this threshold may be more desirable as the target concentration for effective cancer treatment while minimizing adverse effects. Therefore, concentrations of ibrutinib at or below 10 µM may be considered the target concentration for achieving optimal cytotoxicity against the sarcoma (S180) cancer cell line in this study. |

**Reviewer 2**

| **Serial No.** | **Reviewer Comment** | **Response** |
| --- | --- | --- |
| 1 | Title: The authors should consider amending the title to read: “Enhancement of the Oral Bioavailability of Ibrutinib Using a Liposil Nanohybrid Delivery System” | Title amended as per the suggestion and Incorporated in the manuscript |
| 2 | Author details: the author’s details should be consistent and in accordance with the requirements of the journal. | Author details modified as per the journal guidelines and modified in manuscript and highlighted |
| 3 | Spacing: there are numerous spaces omitted between words and between words and references in the manuscript. The authors are advised to do a proof-read of the manuscript to correct these. | The proof reading of the manuscript was thoroughly done multiple times as advised by the reviewer. |
| 4 | Spelling and grammar: there are a few spelling and grammar errors in the manuscript, as well as missing units in presenting the methodology and data. The authors are advised to do a proof-read of the manuscript to correct these. | The grammar has been improved throughout the manuscript as suggested by the reviewer. |
| 5 | Introduction, Paragraph 2, Lines 3-4: The statement ‘…Liposomes with a silica nanoparticle coating…” should be referenced. | Appropriate Reference was cited for the above statement in the manuscript. |
| 6 | Referencing: there are inconsistencies regarding the in-text referencing. The authors are advised to do a proof-read of the manuscript to amend all in-text references to conform to the requirements of the journal. | All the references and in text citations were checked and updated as per journal guidelines. |
| 7 | RP-HPLC methodology: The authors should state if the RP-HPLC method used for quantification of Ibrutinib was validated and should this method be from literature, a reference should be provided. | A reference for the RP-HPLC methodology is provided in the manuscript as per suggestion. |
| 8 | Preparation of ibrutinib nanoliposomes method: The authors of this manuscript have previously published the methodology used for the preparation of ibrutinib nanoliposomes: Ashar F, Hani U, Osmani RAM, Kazim SM, Selvamuthukumar S. Preparation and Optimization of Ibrutinib-Loaded Nanoliposomes Using Response Surface Methodology. Polymers. 2022; 14(18):3886. <https://doi.org/10.3390/polym14183886>. This should be included as a reference in this section. | Suggested Reference added to the reference list and cited in manuscript in section **2.2**. |
| 9 | Equipment details: The details and manufacturer of all equipment used in the preparation and analysis of the formulations in this manuscript should be provided in the methodology section under the respective methods. | The details of the equipment used in the study are included in the manuscript and highlighted. |
| 10 | Low pH stability method: the temperature for this test should be provided. | The Low pH stability experiments were performed at room temperature. Same was included in the manuscript in the section **2.6.2**. |
| 11 | In vitro cytotoxicity study method: the methodology for the culturing of the S180 cells should be provided including the confluency of the culture media prior to the cytotoxicity study. Additionally, the number of sample runs used for the cytotoxicity study should be provided. | The methodology of culturing the S180 cells has been added to the manuscript as advised by the reviewer in the section **2.7**. Total three samples were tested for cytotoxicity evaluation and all the experiments were performed in triplicates. |
| 12 | In-vitro drug release study: Was this study undertaken at pH 1.2 and pH 6.8 to account for transition of the prepared liposils through the gastrointestinal tract? While the exposure of the liposil to pH 2, 4 and 6 has been evaluated in the Low pH stability analysis, the temperature of this study has not been provided and the time period of 30 minutes is not adequate to determine that no release will take place at pH 1.2 and 6.8 in vivo. In vitro drug release data at pH 1.2 and pH 6.8 should therefore be included in the in vitro release study to correlate between the in vitro and in vivo studies. | Suggested data have been incorporated in the manuscript in the section **3.2.2** |
| 13 | Drug release modelling: The equations for the kinetic models employed (Korsemeyer Peppa's, Higuchi's, first-order, and zero-order) to model the release data are known and should be removed. | The equations have been removed from the manuscript as suggested. |
| 14 | In vivo study: the method used for preparation of the ibrutinib suspension should be provided. | The method used for the preparation of the ibrutinib suspension has been included in the manuscript in section **2.10**. |
| 15 | Characterization of ibrutinib liposils: The zeta potential data is present in Table 1; however this has not been mentioned in the manuscript text. | The discussion about zeta potential was included in the manuscript in section **3.1**. |
| 16 | Particle imaging: the quality of the images in Figure 1 is very poor and the scale bars are not legible. | We appreciate the reviewer's feedback regarding the quality of the images in Figure 1 and acknowledge their concerns regarding the legibility of the scale bars. Despite our efforts, the submitted images represent the best quality available to us at this time. However, we recognize the importance of clear and informative visuals in scientific publications. While we regret any inconvenience caused by the quality of the images, we are committed to ensuring the integrity and effectiveness of our scientific communication. We thank the reviewer for bringing these concerns to our attention and for their valuable feedback, which will ultimately contribute to the improvement of our manuscript. |
| 17 | Entrapment efficiency results: the results for the Entrapment efficiency are not described in-text. | The discussion about Entrapment efficiency was included in the manuscript in section **3.1.3**. |
| 18 | FTIR data: references are required for all peak allocations. Additionally, were all the native components of the liposils analyzed in the FTIR study to compare with the FTIR spectra generated for the nanoliposomes and liposils? | Reference for the peak allocation was included in the respective section and cited accordingly in the manuscript in the section **3.1.4.1**. |
| 19 | Differential scanning calorimetry data: references are required for the allocation of the melting point of ibrutinib at 159.12 °C. | Reference was included in the respective section and cited accordingly in the manuscript in the section **3.1.4.3**. |
| 20 | Kinetic modeling: the maximum R2 value of 0.7224 is not high enough to provide a correlation with the models used and therefore determine the mechanism of drug release. The authors are advised to investigate other kinetic models to ascertain the drug release mechanism of the developed liposils. | While the R2 value of 0.7224 may not be as high as desired for definitive correlation with the models used, it still holds valuable insights. I agree that exploring additional kinetic models could provide a more comprehensive understanding of the drug release mechanism in the developed liposils. By incorporating alternative models, we can potentially uncover nuanced aspects of the release process that were not captured by the initial analysis. This iterative approach to kinetic modelling will enhance the robustness of our findings and contribute to a more thorough characterization of the drug delivery system. Thank you for highlighting this aspect, and we will certainly consider your suggestion in our further investigations. |
| 21 | Figure 3, Figure 4, Figure 5 and Figure 6 should include error bars. | The average values and standard deviation are utilized for the graphs throughout the manuscript. The standard error values are very low, hence we avoided the inclusion of error bars in the graphs. |
| 22 | Discussion: there is limited to no discussion of the in vitro and in vivo release data in this section. | Suggested *In vitro* and *in vivo* studies discussion was included in the manuscript and highlighted in section **4**. |
