# Supplementary material for: Enhancement of oral bioavailability of ibrutinib using a liposil nanohybrid delivery system
Source: PLoS One. 2024 Sep 24;19(9):e0310492. doi: 10.1371/journal.pone.0310492 (PMC11421787; doi:10.1371/journal.pone.0310492)
Supplement: Attachment — Submitted filename: Response to reviewer comments.docx [file pone.0310492.s002.docx]

**Response to reviewer comments**

**Reviewer 2**

| **Serial No.** | **Reviewer Comment** | **Response** |
| --- | --- | --- |
| 1 | The in vitro release data provided in Table 2 should be included in the drug release profile in Figure 6b, accounting for residence time in the gastrointestinal tract at pH 1.2, 6.8 and 7.4. This will allow for a better correlation between the in vitro and in vivo data presented | We appreciate the suggestion to include the in-vitro release data from table **2** to figure **6.** We have now integrated these data into figure **6** and accounted for the residence time in the gastrointestinal tract at pH levels 1.2, 6.8 and 7.4. This integration has provided a clearer correlation between the in-vitro and in-vivo data. The revised figure now offers a more comprehensive view of the drug release profile under different physiological conditions. |
| 2 | The referencing of the FTIR data is not complete with the peak designations assigned to the nanoliposomes not referenced | We apologise the oversight regarding the FTIR data referencing. We have now included the appropriate references for the peak designations assigned to the nanoliposomes in the revised manuscript. These references provide a detailed explanation of the characteristic peaks and their significance, thereby enhancing the clarity and completeness of the FTIR data presentation. |
| 3 | The number of sample runs should be included in the Figure captions. Additionally, where error bars have not been included in the images, the standard deviation should be stated in the Figure caption. | We have updated the figures with their respective error bars wherever applicable. We have now replaced the old figures with the new ones. |

We believe these revisions address the reviewers’ comments thoroughly and enhance the overall quality and clarity of the manuscript. Thank you once again for your insightful feedback.
