# Supplementary material for: Enhancement of oral bioavailability of ibrutinib using a liposil nanohybrid delivery system
Source: PLoS One. 2024 Sep 24;19(9):e0310492. doi: 10.1371/journal.pone.0310492 (PMC11421787; doi:10.1371/journal.pone.0310492)
Supplement: Attachment — Submitted filename: Response to reviewer comments.docx [file pone.0310492.s003.docx]

To	31-08-2024

The Editor,

PlosOne Journal.

**Subject: Response to Reviewer and Editor Comments.**

Dear Editor,

We appreciate the time and effort put by the editor and reviewers in providing a fair and comprehensive review of the manuscript. In this letter, we have addressed the comments sent to us and hope that we have answered it as per the standards of the Journal.

Thanks and regards.

Dr S. Selvamuthukumar

Associate professor,

Department of Pharmacy,

Annamalai University,

Annamalai Nagar 608002, India.

Phone: +91 98436 75681

E-Mail ID: smk1976@gmail.com

**Response to reviewer comments**

**Reviewer 2**

| **Serial No.** | **Reviewer Comment** | **Response** |
| --- | --- | --- |
| 1 | The figure caption for Figure 6 should be amended to reflect Figures 6c and 6d. Additionally the label for Figure D in Figure 6 is not legible | We apologise for the oversight. We have now updated the Figure **6** as suggested. The captions for Figure **6C** and **6D** have now been included. |
| 2 | The number of sample runs should be included in the figure captions | The number of sample runs for each experiment performed have now been added as a note in Figure captions |

**Editor comments**

| **Serial No.** | **Reviewer Comment** | **Response** |
| --- | --- | --- |
| 1 | The figure caption for Figure 6 should be amended to reflect Figures 6c and 6d. Additionally the label for Figure D in Figure 6 is not legible | We apologise for the oversight. We have now updated the Figure **6** as suggested. The captions for Figure **6C** and **6D** have now been included. |
| 2 | The number of sample runs should be included in the figure captions | The number of sample runs for each experiment performed have now been added as a note in Figure captions |

We believe these revisions address the reviewers’ and editors’ comments thoroughly and enhance the overall quality and clarity of the manuscript. Thank you for your insightful feedback.
